# Effects of 4‐Oxo‐2‐nonenal on biochemical properties of bovine heart mitochondria

**DOI:** 10.1002/fsn3.2799

**Published:** 2022-03-09

**Authors:** Anand Mohan, Deepak Kafle, Rakesh K. Singh, Yen‐Con Hung

**Affiliations:** ^1^ 1355 Department of Food Science and Technology College of Agricultural and Environmental Science University of Georgia Athens Georgia USA

**Keywords:** 4‐oxo‐2‐nonenal, 4‐Hydroxy‐2‐nonenal, antioxidant properties, lipid oxidation, mitochondria, oxygen consumption

## Abstract

The effects of lipid peroxidation products 4‐Hydroxy‐2‐nonenal (4‐HNE) and 4‐oxo‐2‐nonenal (4‐ONE) were evaluated using bovine heart mitochondria. Oxygen consumption rate (OCR), ultrastructure, antioxidant activity, and membrane permeability were examined to compare their effects on isolated mitochondria from beef cardiac muscle. For the mitochondrial morphology, the final concentration of mitochondria and 4‐ONE or 4‐HNE in the reaction tube were 10 mg/ml and 1 mM, respectively. For the OCR experiment, mitochondria (2.5 mg/ml) were incubated with 0.20 mM ONE or in a Clark electrode chamber at 25°C. Mitochondrial membrane permeability was determined by incubating 0.5 mg/ml of mitochondrial protein with either 0.05 mM ONE or HNE or ethanol control at pH 5.6 and 7.4 at 25°C. Transmission electron microscopy (TEM) revealed that the size of 4‐ONE treated mitochondria at pH 7.4 increased (*p* < .05), as did permeability (*p* < .05), unlike ethanol controls. However, mitochondria incubated with 4‐ONE at pH 5.6 showed a decrease in volume (*p* < .05). Incubating mitochondria with 4‐ONE at pH 5.6 and pH increased oxygen consumption rate 7.4 caused less oxygen consumption than either 4‐HNE treatment or ethanol control. The hydrogen peroxide assay (H_2_O_2_), ferric reducing antioxidant properties (FRAP), and 2,2’‐azinobis (3‐ethylbenzthiazoline‐6‐sulfonic acid) (ABTS.^+^) assays revealed that 4‐ONE is a more potent inhibitor of the endogenous antioxidant system of mitochondria than 4‐HNE (*p* < .05).

## Graphical abstract

4‐ONE can modify structure and function of bovine heart mitochondria more than HNE. ONE is a potent inhibitors of endogenous mitochondrial antioxidant system. ONE represents an important mediator of free radical damage to subcellular structures.

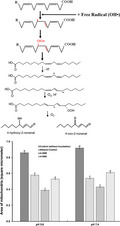

## 1 INTRODUCTION

The biological process of lipid oxidation in postmortem skeletal tissue generates various oxidative products that are considered cytotoxic and highly reactive to other biomolecules. The reactive oxidative species generated in muscle food systems initiate a cascade of chain reactions and become causative agents of food quality deterioration, off‐flavor development, and loss of nutritional value. The secondary lipid oxidation products such as *α*, *β*‐unsaturated aldehydes, and ketones are highly reactive to many biological micro‐ and macromolecules (Witz, 1989). 4‐oxononenal (4‐ONE) and 4‐Hydroxynonenal (4‐HNE) are reactive hydroperoxides generated from polyunsaturated fatty acids (PUFA) like linoleic acid, linolenic acid, and arachidonic acid (Poli & Schaur, 2000; Pryor & Porter, 1990; Schneider et al., 2001; Zhu & Sayre, 2007).

More than 50% of mitochondrial membrane fatty acids are unsaturated (Gutierrez et al., 2002). These membranes are target sites where secondary aldehydes like 4‐HNE and 4‐ONE are produced by lipid peroxidation, and these products can diffuse out of the membranes to modify nucleic acid and proteins (Esterbauer et al., 1991). For instance, oxidation of n‐6 polyunsaturated fatty acids results in lipid hydroperoxides, whose decomposition leads to the formation of 4‐HNE and 4‐ONE, which are protein reactive and cytotoxic (Saito et al., 2011).

4‐HNE is a unique compound with three functional groups: the C = C double bond, the –OH group, and the –CHO (aldehyde group), all of which determine its reactivity with many molecular species, but especially proteins (Chen & Yu, 1994). The reactivity of these aldehydes with amino acids follows the order Cys >> His > Lys > (> Arg for 4‐ONE) (Doorn & Petersen, 2002). 4‐HNE can modify the glucose‐6‐phosphate dehydrogenase, glyceraldehyde‐3‐phosphate dehydrogenase, glutathione S‐transferase, glutathione reductase, aldose reductase, Na^+^K^+^ ATPase, and GLUT‐3 (Uchida, 2003) as well as aminopeptidase (Lee et al., 2010). 4‐ONE can modify proteins ribonuclease A and *β*‐lactoglobulin (Zhang et al., 2003). 4‐HNE incubated with mitochondria isolated from rat brain can decrease membrane fluidity by directly interacting with membrane phospholipids, confirmed by the generation of the fluorescent complex (Chen & Yu, 1994). This can affect mitochondrial morphology and function (Humphries et al., 1998). 4‐HNE inhibits complex I‐linked and complex II‐linked state III respiration in rat brains (Picklo et al., 1999).

The mitochondrial matrix has a high concentration of antioxidant defense enzymes, which can stabilize cellular H_2_O_2_ (Mailloux, 2018). The integrated thiol system within the matrix prevents oxidative damage to protect mitochondria from such reactive species as superoxide and hydrogen peroxide (Murphy, 2012).

Earlier research in science other than meat science has reported that 4‐ONE is more toxic to proteins and neurons than 4‐HNE. No research on food products appears to be available on how much damage 4‐ONE and 4‐HNE can do to structures that affect meat qualities. However, some research does show 4‐HNE affects meat color stability; it can bind covalently with histidine residue within myoglobin and decrease color stability (Alderton et al., 2003). Very little research has assessed how much damage to mitochondrial structure and function is due to the effect of 4‐ONE on meat.

We hypothesize that 4‐ONE, a novel lipid oxidation byproduct, reacts more aggressively than 4‐HNE, influencing mitochondria's structure, function, and defensive ability. Therefore, the objectives of the present study were to compare the effects of 4‐hydroxy‐2‐nonenal and 4‐oxo‐2‐nonenal on the structure, function, and antioxidative capacity of bovine heart mitochondria.

## 2 MATERIALS AND METHODS

In this study, electron microscopy and permeability were used to characterize structural changes, and oxygen consumption was used to characterize mitochondrial function. A radical scavenging assay of the mitochondrial extract was performed to demonstrate whether the endogenous defense system can detoxify these two aldehydes: 4‐HNE and 4‐ONE.

### 2.1 Chemicals

The following chemicals were used in the experiment: bovine serum albumin (BSA), sucrose, tris hydroxymethyl aminomethane hydrochloride (Tris‐HCl), potassium phosphate monobasic (KH2PO4), potassium phosphate dibasic (K2HPO4), ethylene glycol‐bis (*β*‐aminoethyl ether)‐N,N,N’,N’‐tetraacetic acid (EGTA), N‐[2‐hydroxyethyl], piperazine‐N’‐[2‐ethanesulfonic acid], adenosine 5’‐diphosphate (ADP), proteinase K, CaCl_2_, succinic acid, EDTA, and a Bicinchoninic Acid Protein Assay Kit were purchased from Sigma Chemical Co. (St. Louis, MO); sodium succinate was purchased from Fisher Scientific (Fair Lawn, NJ). 4‐HNE and 4‐ONE were purchased from Cayman Chemical Co. (Ann Arbor, MI). Reagents for electron microscopy (glutaraldehyde and osmium tetroxide) were also purchased from Sigma Chemical Co. (St. Louis, MO). ABTS diammonium salt, horseradish peroxidase (≥250 units /mg), phosphate buffer solution (PBS), and ferrous sulfate heptahydrate (FeSO4.7H_2_O) were purchased from Sigma‐Aldrich (St. Louis, MO); H_2_O_2_ solution (30% w/w solution) and 2,4,6‐tri (2‐pyridyl)‐s‐triazine (TPTZ) were purchased from ACROS Organic (Morris, USA). Ferric chloride anhydrous (FeCl_3_) was purchased from Fisher Chemical (NJ, USA).

### 2.2 Mitochondria isolation

A briefly modified procedure by Mohan et al. (2010) was used for isolating the beef heart mitochondrial. Briefly, 50 g of postmortem bovine cardiac muscle was trimmed of visible connective tissue and fat and washed with wash solution before isolating mitochondria. The beef heart tissue was minced using a sterilized stainless‐steel scissor and suspended in 200 ml of isolation buffer (1 mM EGTA, 10 mM HEPES, 250 mM sucrose, pH 7.4). The suspension was gently stirred while slowly adding bacterial Nagarse protease (25 mg/50 g of tissue). The suspension was allowed to incubate for 20 min for the proteolytic reaction to complete. The final pH of the suspended reaction mixture was maintained at 7.4 throughout the incubation time. After completion of the proteolytic reaction, the suspension was gently poured and washed twice with an ice‐cold isolation buffer. Washing was performed to remove residual protease and resuspended to 800 ml of isolation buffer. The suspension was homogenized with three passes using a Kontes‐Duall tissue grinder (Kimbal Chase, Vineland, NJ, USA) and a Wheaton Potter‐Elvehjem tissue grinder (Millville, NJ, USA). The homogenate was centrifuged at 1500 *g* for 20 min at 4℃ temperature. The resulting supernatant was filtered through double‐layered cheesecloth and centrifuged again at 20,000 *g* for 10 min at 4℃ temperature. The pellet was recovered and washed twice with isolation buffer. The mitochondrial pellet was further centrifuged at 15,000 *g* for 5 min at 4℃ temperature to remove extraneous cellular debris and washed twice, and finally suspended in mitochondrial suspension buffer (250 mM sucrose, 10 mM HEPES, pH 7.4). The protein concentration of the mitochondrial suspension was estimated using the bicinchoninic acid assay (BCA, bicinchoninic acid kit, Sigma‐Aldrich, St. Louis, MO).

### 2.3 Effects of 4‐ONE and 4‐HNE on mitochondrial morphology

This experiment was performed to evaluate the influence of 4‐ONE on mitochondrial morphological integrity. The mitochondrial morphological make‐up was investigated using a comparative result of 4‐ONE and 4‐HNE using the method of Ramanathan et al. (2012). The isolated mitochondrial suspension was mixed and incubated with either ethanol or with 4‐ONE (1.0 mM) and 4‐HNE (1.0 mM) in 1.5 ml Eppendorf tubes at 37°C at either pH 5.6 (5 mM phosphate, 120 mM KCl, 30 mM maleic acid) or 7.4 (5 mM phosphate, 120 mM KCl, 30 mM Tris‐HCl). The reaction mixture containing ethanol served as control and used a volume equivalent to that used to deliver the 4‐ONE and 4‐HNE to the reaction mixtures. These experimental conditions were selected and optimized to meet experimental requirements based on previously reported concentrations of 4‐ONE and 4‐HNE in beef skeletal muscles (Ramanathan et al., 2012; Spiteller et al., 2001; Suman et al., 2006).

### 2.4 Transmission electron microscopy of bovine heart mitochondria

Mitochondria were isolated, washed, and processed for the transmission electron microscopy according to Ramanathan et al. (2012) with some modifications. Mitochondrial samples fixed using glutaraldehyde (1.5%) and formaldehyde (1.5%) in a buffer (0.1 M HEPES, 0.08 M NaCl, 3 mM MgCl2). Mitochondrial samples were suspended in low‐temperature gelling agarose (3%; Sigma Aldrich). Suspended mitochondrial samples were then centrifuged at 12,000 *g* at 4°C temperature for 10 min. The pellet was collected, washed, and cut into a 1 mm cube and then treated with a 1% (w/v) osmium tetroxide solution, 0.8% (w/v) potassium ferricyanide, 0.1 M HEPES, and 0.08 M NaCl for 1 h at 4°C. Mitochondrial samples were rewashed with deionized water and dehydrated for 15 min with a graded series of ethanol (30%, 50%, 70%, 95%, and 100%). Final dehydration was performed using 30, 50, 70, and 95% acetone for a minimum of 15 min. Dehydrated samples were then embedded with a mixture of embed 812, Araldite 506, and dodecyl succinic anhydride, and polymerized at 70℃ temperature for 18 h. Final staining was performed using Reynold's lead citrate for 4 min, and samples were prepared for the microscopic view. Images of transmission electron microscopic (100 kV transmission electron microscope) were captured using a JEOL JEM1011 (JEOL USA, Inc., Peabody, MA, USA) at 10,000x magnification. A total of three samples were prepared and used for image analysis. Each image was captured to control sample‐to‐sample variation in terms of image density, and then the average intensity of a region of the resin between mitochondria was measured. Segments were identified for adjacent mitochondria that shared a single boundary, and 80% of this value was used as the segment. Each mitochondrial segment perimeter was selected to measure its area in pixels. Pixel areas were converted into μm^2^ based on the magnification and scan resolution.

### 2.5 Effects of 4‐ONE and 4‐HNE on isolated mitochondrial oxygen consumption

This experiment was conducted to determine if 4‐ONE as compared to 4‐HNE causes adverse effects in mitochondrial oxygen consumption. Mitochondrial samples were treated using a procedure previously described by Purohit et al. (2014) with slight modifications. The mitochondrial oxygen consumption was measured by incubating mitochondria (1 mg/ml) with 0.20 mM of 4‐ONE and 4‐HNE in a Clark electrode chamber (Rank Brothers, Cambridge, U.K.) at 25℃ attached with a PicoLog ADC 20 USB data logger. The data logger was connected to a computer for measuring real‐time data acquisition and storage. Mitochondrial control samples were treated and incubated with a volume of ethanol that was used for treating with 4‐ONE and 4‐HNE for the same time interval. The temperature of the incubation chamber was maintained at 25°C and the cell suspension was kept agitated during oxygen consumption measurements. Mitochondrial oxygen consumption rate was recorded over time by suspending mitochondria at either pH 5.6 (250 mM sucrose, 5 mM KH_2_ PO_4_, 5 mM MgCl_2_, 0.1 mM EDTA, 0.1% BSA, and 20 mM maleic acid) or 7.4 (250 mM sucrose, 5 mM KH_2_ PO_4_, 5mM MgCl_2_, 0.1 mM EDTA, 0.1% BSA, and 20 mM HEPES). The pretreated mitochondrial respiration was measured after adding substrates (8 mM succinate) and ADP solutions to the electrode cell using Hamilton syringes. The pretreated mitochondrial suspension was added until the protein concentration in the cell chamber achieved 1 mg/ml, which is sufficient to exhibit measurable oxygen consumption. The state IV respiration (absence of ADP) was measured using 5 mM of succinate as a substrate. State III respiration was measured by adding ADP (500 μM). The ratio of [state III] to [state IV] was calculated to determine the respiratory control ratio (RCR), which indicates the tightness of the coupling between respiration and phosphorylation. The time of calculation of the slopes varied according to the response given by mitochondria to the substrate and ADP additions.

### 2.6 Determination of mitochondrial membrane permeability

This experiment was conducted to determine if treatment with the treatment of isolated mitochondria with 4‐ONE and 4‐HNE will lead to an increase in mitochondrial membrane permeability (MMP). The MMP is one of the key determinants of physiologically dysfunctional mitochondria. Isolated bovine heart mitochondria were incubated with 4‐ONE and 4‐HNE similar to Ramanathan et al. (2012) with some modification. Mitochondria (0.5 mg protein/ml) were incubated with 4‐HNE (0.05 mM), 4‐ONE (0.05 mM), or ethanol (control) at pH 5.6 (0.1 M MES and 250 mM sucrose) or 7.4 (0.1 M Tris‐HCl and 250 mm sucrose) at 25°C for 50 min. Mitochondria with EDTA (0.3 mm), ethanol, and either CaCl_2_ (0.025 mM) used as positive control for swelling or ATP (3 mM) used as positive control for contraction. Changes in absorbance were measured using UV‐1800 UV‐Vis spectrophotometer (Shimadzu Scientific Instruments, Inc., USA) at 10 min intervals for 60 min (Goodell & Cortopassi, 1998). A decrease in absorbance at 520 nm indicates mitochondrial swelling, and an increased absorbance indicates contraction.

### 2.7 Inhibiting radical scavenging activity of mitochondria

Inhibiting radical scavenging activity of mitochondria was revealed by using the ABTS, H_2_O_2_, and FRAP assays with 4‐HNE and 4‐ONE. Freezing and thawing disrupted mitochondria; mitochondria were then diluted in buffer solution, and the enzymes were recovered to test for radical scavenging (Tonkonogi et al., 2000). Cycles of freezing and thawing of 0.5 g of mitochondria were repeated 2–3 times. The samples were mixed with 10 ml of PBS and vortexed enough to allow the sample to mix uniformly. The mixture was filtered through microfilter paper to collect the filtrate. The collected filtrate was incubated with 0, 15, 30, 45, and 60 μM 4‐HNE or 4‐ONE for 8 min at room temperature. These samples were assayed spectrophotometrically for radical scavenging using FRAP, ABTS.^+^, and H_2_O_2_ assays.

### 2.8 FRAP assay

The FRAP assay was conducted using the procedure described by Benzie and Strain (1996) with minor modification. The FRAP reagent was prepared by mixing solutions of 10 mM TPTZ (in 40 mM HCl), 20 mM FeCl3, and 300 mM acetate buffer (pH 3.6) at a volumetric ratio of 1:1:10. Aqueous solutions of FeSO4.7H2O were prepared at concentrations of 10, 200, 500, and 1000 μM for calibration. Water was used as the blank. Freshly prepared FRAP reagent (900 μl) was mixed with 30 μl of the sample solution, then 90 μl water was added to bring the final dilution of the sample in the reaction mixture to 34:1. The reaction mixture was then incubated in the dark at 37°C for 4 min. The absorbance of the mixture was monitored at 593 nm.

### 2.9 ABTS.^+^ scavenging assay

The ABTS.^+^ radical cation decolorization assay was performed with modifications (Phonsatta et al., 2017; Re et al., 1999). Potassium persulfate (2.45 mM) was mixed with 7 mM ABTS water solution in the dark at room temperature for 16 h. The ABTS.^+^ solution was then diluted with ethanol to obtain an absorbance of 0.70 (±0.01) at a wavelength of 734 nm. Ethanol was used for the control groups. A 100 μl sample was mixed with 900 μl of the ABTS^+^ solution; the mixture was then incubated in the dark at 30°C for 6 min. Absorbance was measured at 734 nm with the spectrophotometer. All experiments were performed in triplicate. All samples and reagents were prepared fresh daily.

### 2.10 H_2_O_2_ scavenging assay

The H_2_O_2_ scavenging assay (Pick & Keisari, 1980; Sroka & Cisowski, 2003) was performed with modifications. Equal amounts of the sample and 0.002% (w/w) H2O2 solution were mixed with 0.8 ml PBS and incubated in the dark at 37°C for 10 min. Assay reagent (1 ml), containing 0.2 mg/ml phenol red and 0.1 mg/ml horseradish peroxidase in PBS, was added and incubated under the same conditions for 15 min. After incubation, 50 μl of 1 M sodium hydroxide was added, and the absorbance of the mixture was measured immediately at 610 nm using the spectrophotometer. H_2_O_2_ solutions at different concentrations (0.0002%, 0.0005%, 0.001%, and 0.002% w/w) were used as the calibration curve.

### 2.11 Statistical analysis

The data were analyzed using Type‐3 tests of fixed effects using the MIXED procedure of SAS (Version 9.1, SAS Institute, Inc., Cary, NC). The experiment was replicated on three separate occasions. Beef heart was a block in a randomized complete block design, and treatments assigned to isolated mitochondria within a heart. Fixed effects were analyzed using a one‐way treatment structure. Analysis of mitochondrial area was performed using approximately 75 individual mitochondrion at multiple locations to minimize variations due to pellet inhomogeneity. Least square means for protected *F*‐tests (*p* < .05) were separated using least significant differences (LSD) and were considered significant at *p* > .05.

## 3 RESULTS AND DISCUSSION

### 3.1 Effects of 4‐HNE and 4‐ONE on the morphology of mitochondria

**FIGURE 1:**
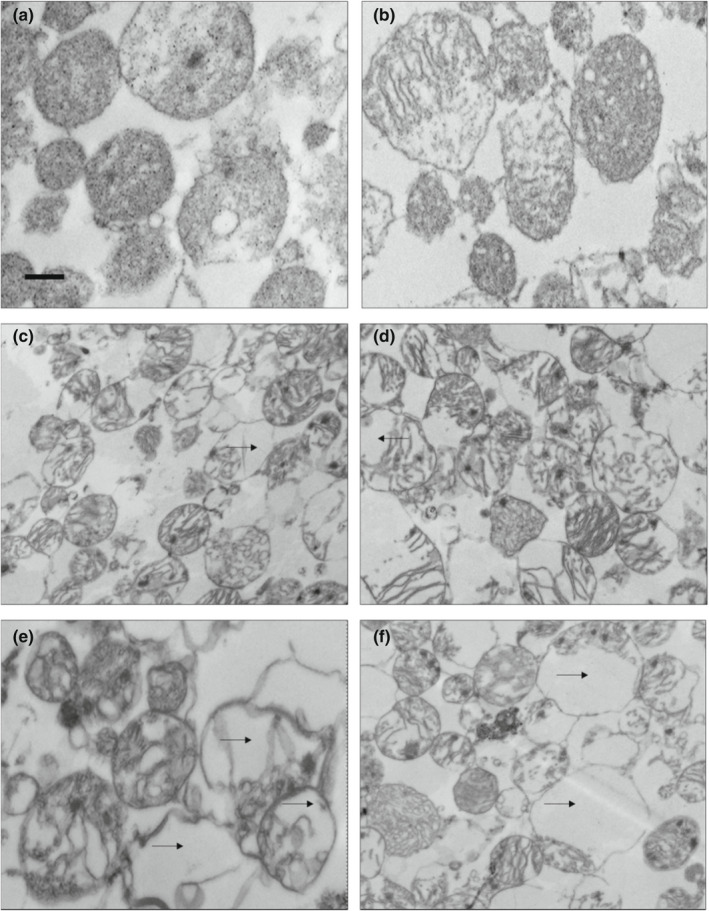
Electron micrographs of mitochondria incubated in (a) pH 7.4 buffer, (b) pH 5.6 buffer, (c) pH 7.4 buffer with 0.4 mM 4‐HNE, (d) pH 7.4 buffer with 0.4 mM 4‐ONE, (e) pH 5.6 buffer with 0.4 mM 4‐HNE, and (f) pH 5.6 buffer with 4‐ONE. The scale bar in panel A corresponds to 500 nm and is the same for panels a–f. Magnification = 10,000×. Arrows in panels c through f indicate mitochondrial vacuolization

**FIGURE 2:**
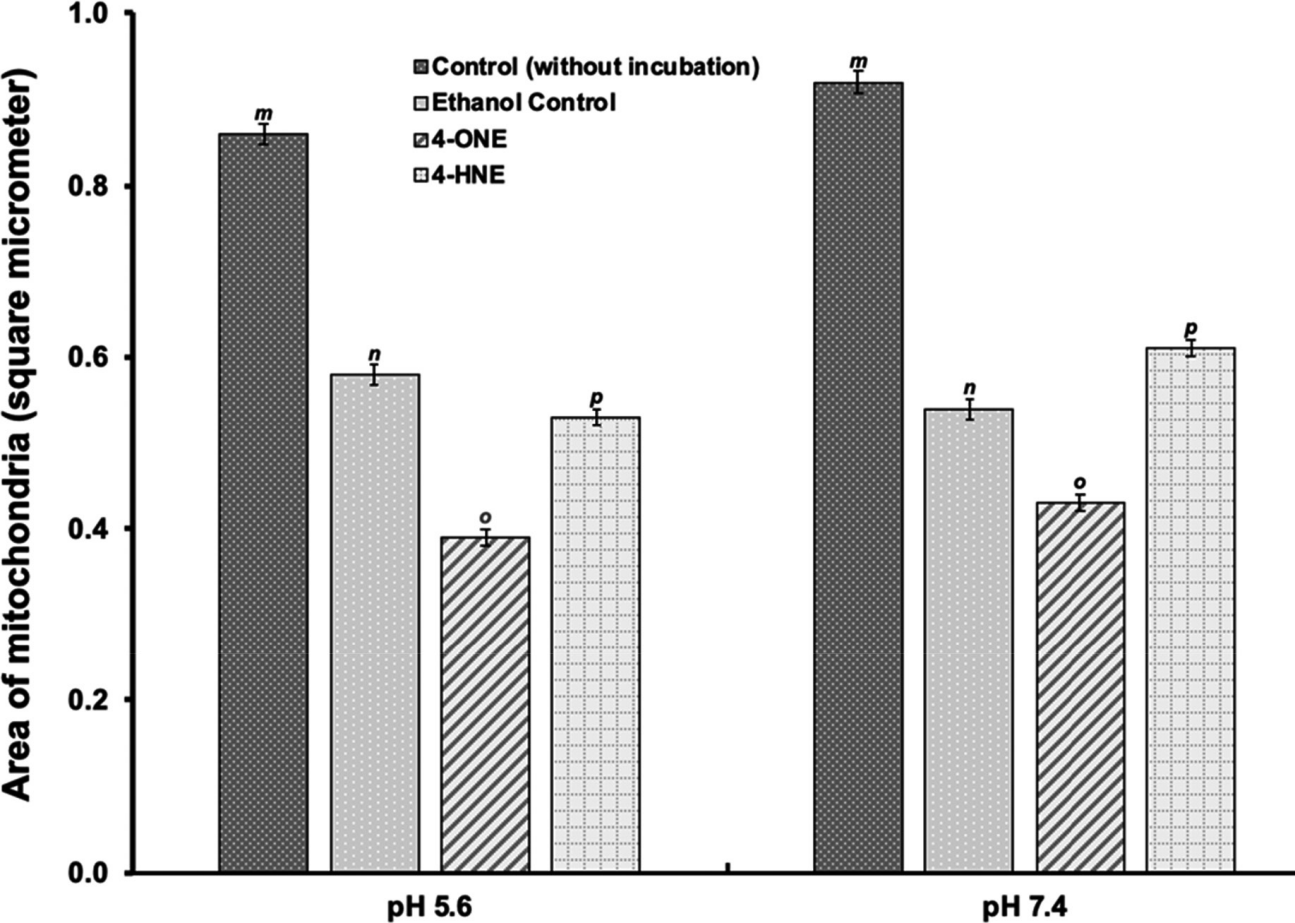
The areas of mitochondria (represented by the length of bars) incubated with HNE and ONE at different pH levels. ^a,b,c^Different letters indicate significant statistical difference at *p* < .05

Figure 1 shows the TEM micrograph of mitochondria treated with 4‐HNE and 4‐ONE. TEM micrograph shows that mitochondrial morphology changed after treatment with 4‐HNE and 4‐ONE. Both pH levels (7.4 and 5.6) responded differently (*p* < .05) to mitochondrial morphological alterations (Figure 2). The mitochondria incubated at pH 7.4 exhibited a higher swollen area (*p* < .05) than those incubated at pH 5.6. Areas of mitochondria were analyzed using Image J software, and the measured areas in square micrometer are presented in Figure 2. The control mitochondria had more intact and consistent structures, whereas those incubated at pH 5.6 with 4‐ONE exhibited disrupted morphological structure. This phenomenon could be explained by the relative toxicity of these aldehydes (Doorn & Petersen, 2002; Lin et al., 2005; Picklo et al., 2011).

Ramanathan et al. (2012) also conducted image analysis of mitochondria isolated from beef heart. Their findings agreed with our results. They found that mitochondria incubated with 4‐HNE at pH 7.4 had more (*p* < .05) area than those incubated with either 4‐HNE at pH 5.6 or ethanol controls. Results of our study showed that the highest area for mitochondria incubated with 4‐ONE at pH 7.4 was 1.071 square micrometers; 4‐HNE incubated at pH 5.6 had an area of 0.448 square micrometers.

Mitochondria can undergo structural changes in response to external stimuli (Pivarova & Andrews, 2010). Lipid peroxidation products like 4‐HNE can modify membrane fluidity by interacting with phospholipids present in mitochondrial membranes (Chen & Yu, 1994). 4‐HNE can induce calcium‐dependent mitochondrial permeability transition, thereby opening the pores and causing the structure to swell (Kristal et al., 1996). Despite the potential of aldehydes to alter membrane fluidity, little research has assessed how aldehydes affect the mitochondrial structure. In a comparative study, 4‐HNE and 4‐ONE both disrupted the normal morphological structure of mitochondria (Gonzalez, 2013). The organelles were incubated with 4‐ONE swelled (area) more than two other treatments (control and 4‐HNE). Other researchers have reported the electrophilic characteristics of 4‐HNE and 4‐ONE differently. Although both are diffusible electrophiles that can reach protein targets from their site of generation (Zhong et al., 2014), 4‐ONE is more reactive to thiols and amines because it makes itself a suicide substrate for metabolic enzymes, which then interact with 4‐HNE, thus increasing the half‐life of 4‐HNE (Doorn et al., 2006). However, 4‐ONE is less reactive than 4‐HNE to Lys/amine conjugate adduction (Sayre et al., 2006).

### 3.2 Effect of 4‐HNE and 4‐ONE on oxygen consumption

Preincubation of mitochondria with these aldehydes (4‐HNE and 4‐ONE) resulted in lower RCR, state III, and state IV oxygen consumption than control samples (*p* < .05). Both aldehydes (4‐HNE and 4‐ONE) significantly affected mitochondrial oxygen consumption at both pH 5.6 and 7.4 (Table 1). The observations presented in this study that 4‐HNE and 4‐ONE decreased OCR (state III and state IV) of mitochondria at pH 5.6 and 7.4 are consistent with findings of Ramanathan et al. (2012). Most notably, in this study, 4‐ONE decreased the OCR more than 4‐HNE (*p* < .05). This characteristic of 4‐ONE induced decrease in the OCR was previously observed in rat brain mitochondria (Picklo et al., 2011), where 4‐ONE was sensitive enough to inhibit succinate‐linked respiration.

**TABLE 1:** Effect of HNE and ONE on oxygen consumption of bovine heart mitochondria

Treatment	pH	State III	State IV	RCR^1^
Succinate	7.4	274.6^a^	95.4^a^	2.9^a^
Succinate + HNE	7.4	124.2^b^	73.2^b^	1.7^b^
Succinate + ONE	7.4	91.5^c^	65.6^c^	1.4^c^
Succinate	5.6	203.7^a^	84.5^a^	2.4^a^
Succinate + HNE	5.6	93.3^b^	71.9^b^	1.3^b^
Succinate + ONE	5.6	70.7^c^	63.9^b^	1.1^c^

Note

Different superscript letters indicate significant statistical difference at *p* < .05.

State III: Oxygen consumption rate (nmol O/min mg mitochondrial protein) of isolated mitochondria in the presence of substrate and ADP.

State IV: Oxygen consumption rate (nmol O/min mg mitochondrial protein) of isolated mitochondria in the presence of only added substrate.

^1^ RCR: ratio between states III and IV respiration.

Although the reactivity of 4‐ONE with mitochondrial protein has been reported in the literature, the most noteworthy is that 4‐ONE reactivity and induced mitochondrial protein modification during oxidative stress are 100 times more than 4‐HNE (Long et al., 2013). The main effects of 4‐HNE are on complex I, complex II, cytochrome *c*, and cytochrome *c* oxidase, thus reducing the OCR of mitochondria (Ramanathan et al., 2012). Humphries et al. (1998) attributed decreased oxygen consumption by rat brain mitochondria as linked to NADH state III respiration because 4‐HNE inactivates complex I. Mitochondria of different tissues respond differently to 4‐HNE (Picklo et al., 1999). The differences in mitochondrial oxygen consumption rates could be attributable to the relative reactivity of 4‐4‐HNE and 4‐ONE to various amino acids.

### 3.3 Effects of 4‐HNE and 4‐ONE on mitochondrial membrane permeability

**FIGURE 3:**
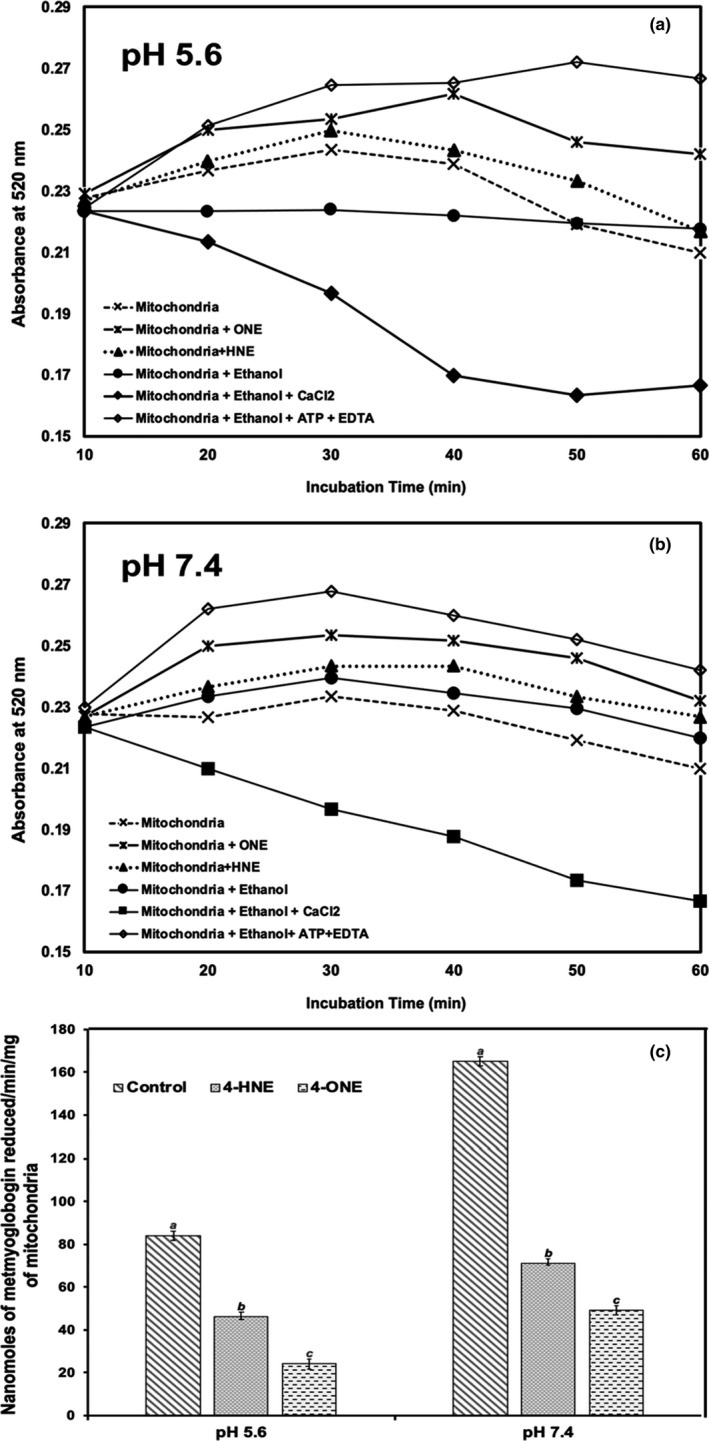
Effect of HNE and ONE on membrane permeability of beef heart mitochondria incubated at 25°C for 50 min at pH 5.6 (a) and pH 7.4 (b). Letters (a, b, c) represents significant differences between ethanol control and mitochondria treated with either HNE or ONE

Several factors affect the permeability of mitochondria. At pH 7.4, the treatment of mitochondria with 4‐ONE caused extensive swelling than ethanol controls at pH 7.4. Comparatively, at pH 5.6, 4‐ONE‐treated mitochondria contracted and did not swell than those at pH 7.4. The occurrence of this phenomenon was supported by measuring the area as well as membrane permeability (see Figures 2 and 3). A decreased absorbance at 520 nm of 4‐ONE‐treated mitochondrial samples at pH 7.4 showed indicated mitochondrial swelling as compared to control samples (*p* < .05). Conversely, mitochondria incubated with 4‐ONE at pH 5.6 had decreased area (contraction) and permeability, shown by increased absorbance at 520 nm.

Similarly, 4‐HNE at pH 7.4 influenced mitochondrial membrane pore opening, which also resulted in mitochondrial swelling (decreased absorbance at 520 nm) (Ramanathan et al., 2012). Prooxidants should trigger mitochondrial permeability (Kushnareva & Sokolove, 2000), which is induced by several cellular toxins, including oxidants like hydroperoxides, Ca^2+^, and neurotoxins (Yang & Cortopassi, 1997). 4‐ONE increases permeability due either to mitochondrial damage or inducing mitochondrial permeability transition (MPT) (Goodell & Cortopassi, 1998). Soon after mitochondria are isolated, they begin to swell, lose the ability to phosphorylate, and release matrix contents if Ca^2+^ ions are not excluded from the suspension (Zoratti & Szabo, 1995).

### 3.4 Effects of 4‐HNE and 4‐ONE on antioxidant activity

**FIGURE 4:**
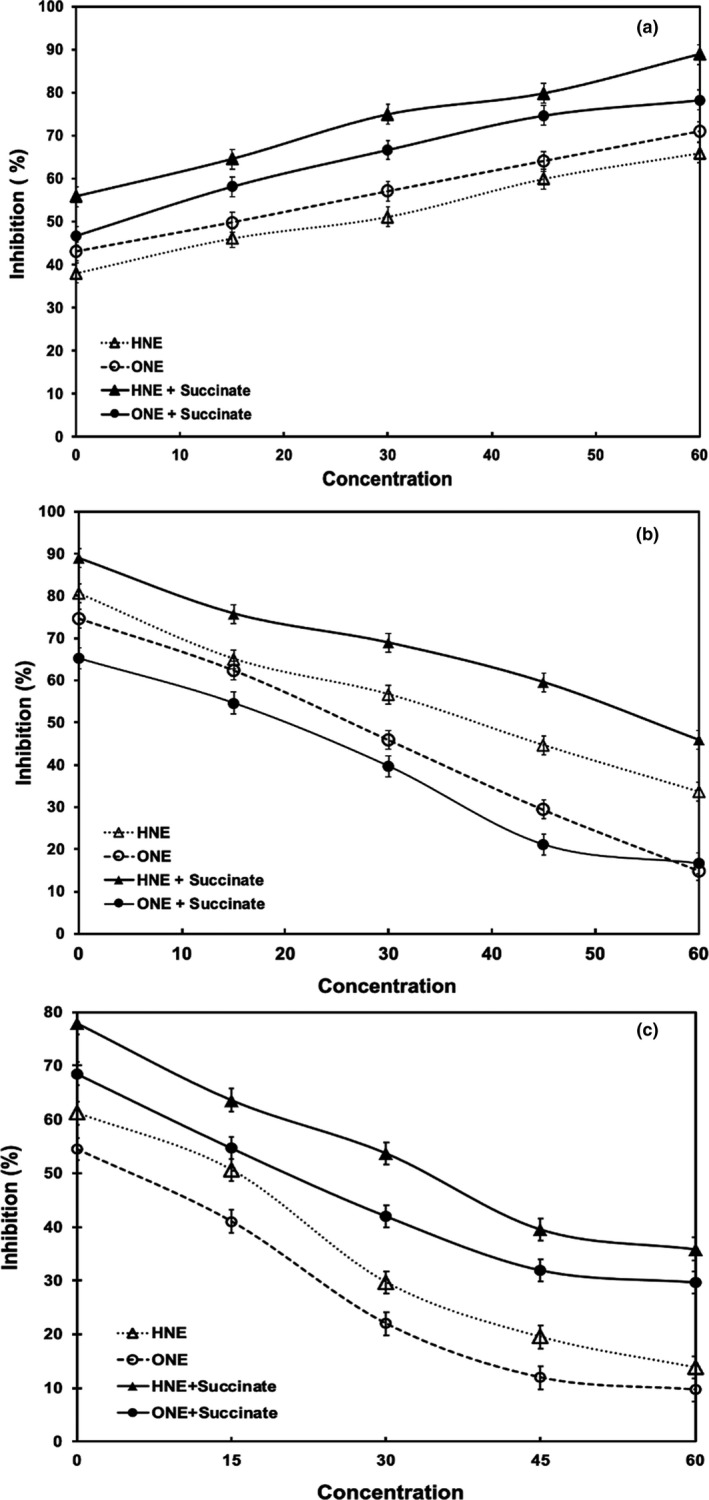
Inhibition of radical scavenging activity of bovine heart mitochondria by HNE and ONE (a) H_2_O_2_ Scavenging assay (b) FRAP Assay (c) ABTS.^+^ scavenging assay

Mitochondria contain an antioxidant defense mechanism to quench ROS, but the entire defense system is not used up in clearing these free radical species (Mailloux, 2018). The mitochondrial antioxidant defense system includes both enzymes like SOD, glutathione peroxidase, and glutathione reductase and nonenzymatic substances like glutathione, *α*‐tocopherol, and coenzyme Q (Paradies et al., 2001). The ABTS.^+^ radical scavenging, FRAP, and H_2_O_2_ scavenging assays showed the inhibition effect of 4‐HNE and 4‐ONE on the defense system (Figure 4). All assays indicated that 4‐ONE inhibited the antioxidant defense system more than 4‐HNE (*p* < .05).

The antioxidant activity of compounds can be expressed as the effective concentration for obtaining a 50% response (EC_50_) or the inhibitory concentration at 50% response (IC_50_) (Zhang & Akoh, 2019). In our study, IC_50_ (the concentration of tested aldehydes in the antioxidant system to reduce free radicals to 50% of initial concentration) was used to quantify the inhibition of antioxidant activity. Reinheckel et al. (1998) suggested that mitochondria could generate sufficient secondary aldehydes to deplete GSH levels. Chen and Yu (1994) reported that incubating 10–400 μM 4‐HNE causes the 4‐HNE adduct to form with cytochrome oxidase, which in turn inhibits enzyme activity. Although 4‐HNE is a modifier of mitochondrial enzyme activity, no research has assessed the reactivity and effect of 4‐ONE on the structure, function, and antioxidant capacity of mitochondria. Electron microscopy and the permeability assay clearly show that 4‐ONE caused mitochondria structural changes and decreased oxygen consumption and mitochondrial function.

Although 4‐HNE and 4‐ONE can modify amino acid nucleophiles, their reactivity differs both qualitatively and quantitatively (Doorn & Petersen, 2002). In our experiment, 4‐ONE inhibited antioxidants more than 4‐HNE. This could be due to the higher reactivity of 4‐ONE to thiol nucleophiles, which is an essential component of glutathione (GSH) (Lin et al., 2005). Our findings suggest 4‐ONE causes irreversible enzymatic inhibition while 4‐HNE does not as shown by mitochondrial respiration. Doorn et al. (2006) found that human mitochondrial aldehyde dehydrogenase lost 10% of its enzyme activity with 4‐HNE but more than 90% of its activity with 4‐ONE. This indicates that 4‐HNE enzyme inhibition is reversible, but 4‐ONE inhibition is mostly irreversible (Doorn et al., 2006).

A study using yak muscle suggested that ROS increased mitochondrial lipid peroxidation and suppressed the activities of SOD, CAT, and GSH‐Px (Wang et al., 2018). Prooxidants are detoxified by the endogenous defense system to balance toxic compounds generated through metabolic activity in the tissues.

In summary, this study revealed that both 4‐HNE and 4‐ONE can influence the structure and function of beef heart mitochondria. In comparison to 4‐HNE, however, 4‐ONE disrupted more of the morphological structure of mitochondria at pH 5.6, although the 4‐ONE‐treated mitochondria were most swollen at pH 7.4, as shown in the higher permeability after incubation with 4‐ONE. Similarly, after preincubation of mitochondrial extracts with these aldehydes, 4‐ONE inhibited the antioxidant system more than 4‐HNE. The results of this study confirm that 4‐ONE is a more toxic biomarker of lipid peroxidation product than 4‐HNE.

## CONFLICT OF INTEREST

On behalf of all authors, the corresponding author states that there is no conflict of interest.

## Data Availability

Data sharing not applicable to this article as no datasets were generated or analysed during the current study.
